# Supporting carers to improve patient safety and maintain their well‐being in transitions from mental health hospitals to the community: A prioritisation nominal group technique

**DOI:** 10.1111/hex.13813

**Published:** 2023-07-08

**Authors:** Sarah McMullen, Maria Panagioti, Claire Planner, Sally Giles, Ioannis Angelakis, Richard N. Keers, Catherine Robinson, Yu Fu, Judith Johnson, Natasha Tyler

**Affiliations:** ^1^ Centre for Primary Care and Health Services Research, Division of Population Health, Health Services Research and Primary Care, Faculty of Biology, Medicine and Health The University of Manchester Manchester UK; ^2^ NIHR Greater Manchester Patient Safety Translational Research Centre The University of Manchester Manchester UK; ^3^ NIHR School for Primary Care Research The University of Manchester Manchester UK; ^4^ Department of Primary Care and Mental Health The University of Liverpool Liverpool UK; ^5^ Division of Pharmacy and Optometry, Centre for Pharmacoepidemiology and Drug Safety The University of Manchester Manchester UK; ^6^ NIHR Greater Manchester Patient Safety Translational Research Centre Manchester Academic Health Science Network Manchester UK; ^7^ Suicide, Risk and Safety Research Unit Greater Manchester Mental Health NHS Foundation Trust Manchester UK; ^8^ Social Care and Society, School of Health Sciences The University of Manchester Manchester UK; ^9^ Population Health Sciences Institute Newcastle University Newcastle UK; ^10^ School of Psychology University of Leeds Leeds UK

**Keywords:** care transitions, carers, discharge, inpatient services, mental health, nominal group technique

## Abstract

**Introduction:**

Carers of people with mental illness may face distinct challenges, including navigating fragmented health and social services during discharge from mental health hospitals. Currently, limited examples of interventions that support carers of people with mental illness in improving patient safety during transitions of care exist. We aimed to identify problems and solutions to inform future carer‐led discharge interventions, which is imperative for ensuring patient safety and the well‐being of carers.

**Methods:**

The nominal group technique was used which combines both qualitative and quantitative data collection methods in four distinct phases: (1) problem identification, (2) solution generation, (3) decision making and (4) prioritisation. The aim was to combine expertise from different stakeholder groups (patients, carers and academics with expertise in primary/secondary care, social care or public health) to identify problems and generate solutions.

**Results:**

Twenty‐eight participants generated potential solutions that were grouped into four themes. The most acceptable solution for each was as follows: (1) ‘Carer Involvement and Improving Carer Experience’ a dedicated family liaison worker, (2) ‘Patient Wellness and Education’ adapting and implementing existing approaches to help implement the patient care plan, (3) ‘Carer Wellness and Education’ peer/social support interventions for carers and (4) ‘Policy and System Improvements’ understanding the co‐ordination of care.

**Conclusion:**

The stakeholder group concurred that the transition from mental health hospitals to the community is a distressing period, where patients and carers are particularly vulnerable to safety and well‐being risks. We identified numerous feasible/acceptable solutions to enable carers to improve patient safety and maintain their own mental wellbeing.

**Patient and Public Contribution:**

Patient and public contributors were represented in the workshop and the focus of the workshop was to identify the problems they faced and co‐design potential solutions. Patient and public contributors were involved in the funding application and study design.

## 1 BACKGROUND

Informal carers provide unpaid help to a friend or family member needing support. According to the 2021 census, more than 5.7 million people are estimated to be informal carers in the United Kingdom.^1^ Carers Week in their 2022 report have estimated that the number of informal carers in the United Kingdom could be as high as 10.6 million.^2^

A total of 1.5 million people care for someone with a mental illness.^3^ Patient safety policies increasingly encourage carer involvement in reducing patient harm. One recent study found that carers who intensively engaged during hospital care provided patients with greater protection, but typically experienced negative consequences for themselves. The authors concluded that carer involvement in patient safety needs to be better understood, especially from the carers' perspective and negative consequences for carers need to be mitigated by practice improvements that value their contributions.^4^

This is especially true for carers of people with mental illness who may face distinct challenges because mental health problems are not seen. They might experience what is called ‘hidden caring’ where family carers may not recognise themselves as carers due to which they are less likely to access support.^5^ However, they might be providing all sorts of help including emotional support, encouragement, practical help with daily tasks and advocacy.^6^ Carers may have serious concerns about the safety of the people that they care for and experience stigma. They may feel responsible/guilty when the patient's health deteriorates or when patient safety incidents occur.^7^ These feelings are especially heightened when they care for people with mental illness.^8^ Thus, it is not surprising that caring is seen as a chronic stress experience.^9^ Carers often experience psychological distress symptoms, including anxiety and depression, and may fail to meet their own health needs or make unhealthy lifestyle choices.^7^

All these challenges and especially those related to patient and carer safety, might worsen during the transition from inpatient mental health services to the community.^10^ Inpatient mental health settings pose unique challenges for patient safety which also influence the discharge process including interpersonal violence, coercive interventions, safety culture, harm to self and safety of the physical environment.^11^ Discharge is often described as a chaotic time with multiple threats to patient safety. For example, the weeks after discharge have been associated with numerous adverse outcomes, including self‐harm, medication safety incidents, suicide and violence.^8^ Adverse social outcomes which include loneliness and homelessness have also been reported.^12^ Systems feel fragmented to many carers whereby social and clinical services seem funded and operated separately and miscommunication is common.^13^ Carers must coordinate and navigate fragmented health and social services when their loved one is discharged from mental health hospitals.^7^ They are often the individuals that must advocate for the patient and act as a ‘boundary spanner’ between fragmented services.^14^ Fragmented services can be defined as a lack of coordinated care between health and social care for patients and carers.^15^ Fragmentation of services is linked to the quality of care provided and poor clinical and social care outcomes.^16^ Carers transfer important information between services and are sometimes the only constant in the patients' health and social care network.^17^ Having effective social support (often provided by carers) is thought to reduce the likelihood of adverse events, such as suicide postdischarge.^18^ The important role of carers during transitions of care has been highlighted during the pandemic.^19^ This was because large numbers of patients were discharged from inpatient mental health services while access to community services was limited.^20^

A recent systematic review found only 12 carer involvement interventions to improve patient outcomes (e.g., readmissions) during mental health transitions.^21^ From the 12 interventions, the interventions which supported carers across the full care pathway were the most promising.^21^ A recent study involving patient engagement activities found that many carers had concerns about the safety of the person they cared for and their own mental health and safety during care transitions.^8^ They described feeling unsupported, lonely and depressed in the community, and were unable to optimally support their loved ones.^8^ Moreover, carers for those with mental illness felt they were not involved in discussions and decisions around discharge and had insufficient information about available support services in the community. These feelings were amplified during the Covid‐19 crisis.^22^ Another study found that competence and listening skills of staff members, concerns about waiting times, staffing levels and overall resourcing of services were key safety issues for mental health service users, carers and professionals. However, that study did not identify solutions and did not focus specifically on care transitions.^23^

Supporting carers of people with mental illness during transitions of care is imperative for ensuring patient safety and the well‐being of carers themselves.^24^ Patient safety and carer wellbeing challenges during discharge from mental health hospitals span social care, primary care, secondary care and public health with carers being expected to skillfully interact with the multiple professionals across diverse disciplines who are involved in the care of their loved ones. Therefore, interdisciplinary lenses are needed to develop multiagency solutions.^8^ To address this, in the present study, we conducted a workshop that brought together patients and carers as well as expertise academics (some with clinical/social care backgrounds) within primary care, mental health, social care and public health. The work aimed to identify problems and solutions to inform future carer‐led patient safety interventions after hospital discharge as well as interventions to support the carer's well‐being during this challenging time.

## 2 METHODS

### 2.1 Study design

The widely used nominal group technique (NGT) was used which combines both qualitative and quantitative data collection methods.^25^ Delbecq et al. proposed the initial model for NGTs and set out four distinct phases: (1) problem identification, (2) solution generation, (3) decision making and (4) prioritisation, implementation and intervention development.^26^ Phases 1–3 were conducted as one online workshop event and phase 4 was conducted separately using online survey technology. The study team brought together stakeholders from health services and the community to identify problems and coproduce solutions for feasible interventions.^27^

#### 2.1.1 Phase 1 problem identification

Participants were asked first in smaller breakout rooms to broadly identify problems that affect safety of people discharged from mental health services and the well‐being of carers. The results of each breakout room were fed back to the full group.

#### 2.1.2 Phase 2 solution generation

Participants within each breakout room were asked what services could do to solve the problems initially highlighted in phase 1. All solutions identified within each breakout room were then discussed with all participants as one group.

#### 2.1.3 Phase 3 decision making

Participants decided which of the potential solutions should be prioritised considering also their feasibility (*the possibility that can be achieved is reasonable*) and acceptability (how much they like the idea) leading to a reduction to the list generated in phase 2.

#### 2.1.4 Phase 4: Prioritisation, implementation and intervention development

The study team reviewed and merged solutions to create a list that is manageable for participants to understand and rank. A Qualtrics survey^28^ was designed for the ranking exercise. Each solution was classified into four main themes, this was done through discussion between the immediate project team (S. M., M. P. and N. T.) highlighting and discussing what each intervention primarily addresses. Participants were asked to rank each solution (from the least to the most important) within each theme by feasibility and acceptability. Cumulative scores were generated by reversing the ranking scores and adding them together across participants. The focus of the analysis was on the top three solutions ranked. The survey was sent to participants by email after the workshop.

Phases 1–3 of the workshop took place as one online session via Zoom hosted by the lead researcher (N. T.). The workshop started with an introduction to the project scope including the background, layout of the session and prompt questions. Twenty‐eight participants took part in the workshop and were split into four breakout rooms. Each breakout room had a facilitator from the University of Manchester with experience in facilitating NGTs. All four groups were evenly distributed and included patients, carers, academics and professionals. As a part of the phase 4 ranking exercise on Qualtrics, participants were asked to fill out their demographic data: age, ethnicity, gender, stakeholder group (patient, carer, practitioner, academic, other), professional role/job title and the number of breakout room (1, 2, 3, 4).

### 2.2 Participants' recruitment and eligibility

Participants were recruited via already established contacts within the study team and through social media. Links with relevant universities and third‐party groups were also established. To help recruit people from underserved communities and maximise the relevance of the findings to the community, the recruitment approach was carefully designed to be inclusive (format/type/language used on adverts) of patients/carers with diverse backgrounds to capture the voices of research underserved groups.

The eligibility criteria were broad so that patients/carers were not excluded based on demographic factors, social and economic factors, and factors related to health status and health conditions as described in the NIHR‐INCLUDE guidance.^29^

The eligibility criteria for the NGT were the following:

1. 18 years and older AND
2. past mental health inpatients OR
3. carers, such as relatives OR
4. academics (with primary care, social care or public health background).

The eligibility of interested participants was further confirmed by the study team using the following screening questions. To take part in the workshop participants would have to return a completed screening pro‐forma with the following items:

1. Your primary stakeholder group (are you a patient, informal carer, professional‐state job title).
2. Do you have direct experience of discharge from inpatient mental health services (Yes OR No).
3. Would you prefer to be in a patient‐/carer‐only group or a mixed group with other stakeholders (question presented only to carers).
4. Please provide 3–4 sentences about yourself and why you would like to take part in the workshop.

Once eligibility was confirmed, participants were sent the information sheet, topic guide (outlining the itinerary for the workshop and discussions) and a consent form to sign and return to the study team. Once the consent form was signed and returned, participants were sent the Zoom link for the workshop. The NGT workshop took place on 15 June 2022 and was approximately 3 h long with scheduled breaks. Participants received £25 per hour for taking part in the workshop, in line with the INVOLVE guidelines.^30^

### 2.3 Data sources

Relevant data from phases 1–3 were collected by the host and breakout room facilitators in the form of hand‐written notes. Further to this, the whole workshop including the breakout rooms was audio recorded. For phase 3, anonymous rankings were collected using Qualtrics and were analysed independently by the two researchers within the study team (S. M., N. T.). Handwritten notes collected during phases 2 and 3 (solution generation) were used to provide context towards the ranking exercise (phase 4).

### 2.4 Analysis

For the analysis, we used the Qualtrics data generated in the survey. We asked participants to rank the solutions within each theme by feasibility and acceptability (1 being the most feasible/acceptable and the last as the least). We reverse‐coded the data which gave us the cumulative ranking from the most acceptable/feasible solution (highest number) to the least (lowest number).

## 3 RESULTS

### 3.1 Demographics

Twenty‐eight participants took part in the workshop and 17 of them (61.8%) also completed the ranking survey on Qualtrics sent by email after the workshop. During phases 1 and 2 of the workshop (Zoom event), breakout rooms one to three were an equally distributed mix of stakeholder groups. Breakout room four consisted only of carers because some carers had stated that they would prefer to be in a separate group during the screening stage.

Table 1 describes the demographics of the 17 participants who took part in the workshop and completed the online survey. We did not collect demographic data from participants who did not complete the survey. Participants were a mixed group of patients (29.4%, *n* = 5), carers (35.2%, *n* = 6) and academics (35.2%, *n* = 6), many academics also had a clinical/social mental health professional background. Two social care professionals and one academic also had lived experience as a carer. The mean age was 43 years (26–55) exact age was reported; however, we presented it categorically to increase anonymity. Eleven participants were female (64.7%), 10 participants identified as White British (White or British) ethnicity (58.8%), 2 (11.8%) Mixed and 1 person (5.9%) identified as Black Caribbean, Greek, Asian British and British Pakistani, 1 (5.9%) did not disclose.

**Table 1:** Demographics data of all participants taking part in phase 1, phase 2 and phase 3 of the workshop.

Age group	Ethnicity	Gender	Stakeholder group	Professional role/job title
26–35	White British	Female	Informal Carer	
36–45	Asian British	Female	Patient	
36–45	White British	Male	Academic	Academic in Pharmacy
46–55	British Pakistani	Female	Informal Carer	
46–55	Mixed	Female	Patient	
46–55	White British	Female	Informal Carer	
26–35	White British	Male	Informal Carer	
26–35	White	Female	Patient	
46–55	British	Female	Patient	
46–55	British	Female	Patient	
36–45	Greek	Male	Academic	Research Associate
36–45	Mixed	Male	Academic	Expert by Experience
46–55		Male	Academic	Carers Lead
36–45	White British	Female	Academic	Lecturer
46–55	White British	Female	Informal Carer	
46–55	Black Caribbean	Male	Informal Carer	
36–45	White British	Female	Academic	Research Fellow

### 3.2 Phase 1: Problem identification

Several distinct patient safety and carer well‐being concerns were identified by the four breakout rooms. One commonly identified concern across the breakout rooms was a lack of support for carers with regard to their mental health and difficulties navigating the discharge process (such as a lack of awareness on how to best support the patient). Table 2 lists the highlighted safety concerns for patients discharged from mental health services and carer well‐being concerns by all four groups.

**Table 2:** Problems identified by participants during phase 1.

1. *Difficulty navigating discharge process*: The discharge process is difficult for carers to know how to support patients (e.g., confidentiality)
2. *Difficulty navigating the transition between CAMHS and adult services*: Carers feel they are expected to take responsibility
3. *Difficulties if carers aren't family*: If not family members, this might affect the way carers are seen by mental health professionals—creates confusion with professionals (uncertainty and delays and the involvement)
4. *Carers guilt*: Periods of deterioration (close to section)—when the patient has no insight of becoming more unwell, if a carers is involved in this, they can feel very guilty
5. Lack of immediate support during recovery
6. *Carers have insufficient knowledge about illnesses*: Carers need to be educated about the specific illness/diagnosis
7. *Carers difficulty managing work and caring repsonsibilties*: Carers who work are feeling stressed to leave people with mental illness alone and risk their safety/people might feel abandoned
8. *Strain on the family dynamic*: A child might put a strain on the whole family, and it is difficult to support with the whole family and the relationship
9. *Difficulties faced by people without carers*: People without carers, e.g., widowed or have no contact with family
10. *Insufficient carer involvement preadmission*: Not much involvement of carers very early on before admission
11. *Carer's own mental health and wellbeing are affected*: For example, higher levels of stress, anxiety, depression and other mental health effects are common
12. *Patients health deterioration waiting for service availability*: Health deterioration due to lack of resources, beds, carer staff at the hospitals.
13. *Emotional impact of improper hospital discharge* for patient and carers (stress, anxiety, anger)
14. *Lack of carer engagement policies at the hospitals*, e.g., carer charter, carer strategy, MH strategy at the NHS acute trust.
15. Risk of self‐harm—suicide and risk management
16. Human resource shortages
17. Carers feeling isolated
18. *Insufficient co‐ordination of care with primary care and wider community services* (which could include families)
19. *Insufficient access to wider services and support* (such as community assets to support wellbeing, work, etc.), especially in the context of people who may face major barriers to accessing services themselves

Abbreviations: CAMHS, child and adolescent mental health services; MH, mental health.

### 3.3 Phase 2: Solution generation

There were many potential ideas generated across the four breakout rooms. These ranged from specialist teams to support patients and carers when they are transitioning back into their home, to family therapy and training techniques for patients and carers. Techniques suggested included behavioural activation and problem‐solving skills to manage everyday stressors.

### 3.4 Phase 3: Decision making

After a group discussion with regard to the potential solutions highlighted in phase 2, the list was combined (solutions that were very similar grouped together) into 34 potential solutions generated across the four breakout rooms. Potential solutions ranged from improving access to services to family therapy and carer wellness interventions. Table 3 lists the 34 potential solutions generated.

**Table 3:** Potential solutions were generated during phase 3 of the workshop.

(1) Improving access to services
2) Quick access to therapies
(3) *Dedicated family liaison worker*: To act as a bridge between hospital and home, including checking the safety of home to return to, etc.
(4) Working with carers to develop *a full pathway of mental health support*, from emotional support and prevention to everyone stepping up to more intensive support for those who need it. Perhaps delivered by carers champions within services like IAPT.
(5) Having *collaborative discharge planning* from the hospital with the patient, family worker (as suggested above) and carer. Perhaps adapting existing approaches such as activity scheduling/BA type approach to help the patient plan what they need to do and what they need help with and plan where that help will come from, e.g., cooking, bills, cleaning, medication, etc.
(6) More *education for families* on conditions
(7) Tailored *peer support intervention* for carers, in a format that is convenient to the people involved, that may be face to face, remotely, telephone, etc.
(8) *Ring‐fenced time* at the end of appointments for staff to speak to carers
(9) *Home environment check* at admission/predischarge
(10) *Financial grants for travel expenses* for carers
(11) *Improving inpatient experience for carers*, i.e., carers resource pack, open communication and consistent feedback between acute staff and carers
(12) *Practical risk management guidance* for acute to community transition
(13) Carers' needs assessments assessing the needs of carers at discharge
(14) *More clarity on co‐ordination of care*: Who should be doing it, what works, what models are more or less effective
(15) *Practical approaches to enable the delivery of person‐centred care*: Person‐centred care is something that is well recognised and understood but remains extremely difficult to actually deliver, an intervention that provides a practical way of delivering this would be useful
(16) *Better implementation of existing solutions*: A lot of the issues flagged are areas where there is quite a good understanding of what needs to happen, but less in terms of how we ensure it is delivered in an effective and efficient way
(17) *Package of support* based on both carer (if there is one) and the patient's needs
(18) *Self‐care and coping strategy training for patients*: Patients being educated and receiving basic training in self‐care and important coping strategies, including behavioural activation and problem‐solving skills which can help them manage their everyday stressors
(19) *Psychoeducation around mindset*: The risks of being focused only on the negatives; learn techniques to switch off to positives
(20) Mindfulness‐based meditation, and relaxation techniques for carers
(21) *Carers illness education*: Carers receive basic training about specific illnesses and being educated about the importance of more generic stressors that other people may face
(22) *Transitional discharge model*: Peer support to individuals who are looking for additional support following a discharge.
(23) *The process to improve communication between carers and staff*: Carer staff/leads at hospitals to engage and involve carers in discharge and carer pathways
(24) *Joined up services working in conjunction* not ringfencing funding
(25) *Specialist MDT discharge team*: Team of doctors, nurses and care staff, etc., who handle discharge for patients and carers till they are discharged and settled in the home.
(26) *Postdischarge follow‐up*: Weekly check‐in with both patient and carer for the 12 weeks following discharge to ensure things are going well
(27) *Carers wellbeing support*: Support for carers so they don't become unwell themselves inc. advice on support groups, etc.
(28) Carers discharge planning involvement involving carers in discharge planning
(29) *Start support planning/information capture at admission*: Identify who the support network is for the patient, and how much involvement can be provided and this can then help with the discharge
(30) *Improvement of CAMHS—adult transition*: Make the transition from CAMHS to adult services age later or the services to work together for some time
(31) *Family therapy*: An evidence‐based approach to treating adolescents that focuses on intervening directly with family members to repair relationships and addressing challenges encountered by adolescents and caregivers in key extrafamilial systems
(32) *Talking services for carers*: Having people that carers can speak too. Patient to talk to someone when a carer is not there
(33) *Improving the importance of carer support in policy and practice*: NHS services and hospital trusts are to elevate supporting carers to the same importance as safeguarding
(34) *Improving peer communication between carers*: Carers need to connect with each other and recognise their role. Time for self‐care for patient

Abbreviations: BA, behavioural activation; CAMHS, child and adolescent mental health services; IAPT, improving access to psychological therapies; MDT, multidisciplinary team.

### 3.5 Phase 4: Prioritization, implementation and intervention development

**Figure 1:**
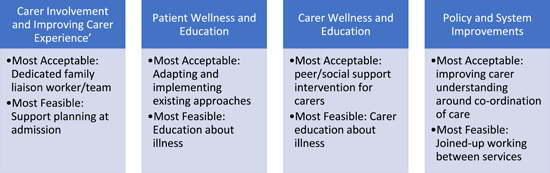
The solutions in each category deemed most acceptable and feasible by the group.

The list of 34 potential solutions was reduced to 20 solutions by the research team, many of the potential solutions were similar so were combined. Workshop participants were then sent a Qualtrics survey which comprised four main themes and were asked to rank each of the solutions within each theme by feasibility and acceptability. The four main themes (carer involvement and improving carer experience, patient wellness and education, carer wellness and education, policy and systems improvements) and the solutions within these themes were derived from the discussions in phase 3. Participants felt it too difficult to rank such a large list of solutions, we collectively so decided to create four smaller themes, based on the primary problem the solutions aim to address. Table 4 lists out the four themes and the solutions within each theme including the cumulative ranking (by reversing the ranks and adding them together across participants) for each solution within the themes, Figure 1 outlines the most feasible (*the possibility that can be achieved is reasonable*) and acceptable (how much they like the idea) and acceptable solutions within each theme.

**Table 4:** Themes and solutions for participant cumulative ranking by feasibility (F) and acceptability (A) (phase 3).

Theme 1: Carer Involvement and Improving Carer Experience	A	F	Theme 2: Patient Wellness and Education	A	F	Theme 3: Carer Wellness and Education	A	F	Theme 4: Policy and System Improvements	A	F
Dedicated family liaison worker or team for discharge to act as a bridge between the hospital and home, including checking the safety of the home when the patient returns. Weekly check‐in with both patient and carer for the 12 weeks following discharge to ensure things are going well.	46	30	Adapting and implementing existing approaches such as activity scheduling to help the patient plan from, e.g., cooking to bills, etc. Ensuring person‐centred care is delivered in an effective and efficient way.	50	55	Basic training/education for carers and patient families about specific illnesses and being educated about the importance of more generic stressors that other people may face.	40	38	Checking the home environment is checked at admission/predischarge.	51	58
Start at admission: identify who the support network is for the patient, and how much involvement can be provided and this can then help with the discharge.	44	51	Patients should receive basic training in self‐care and important coping strategies to manage their everyday stressors.	42	58	Carers needs assessment and mindfulness‐based meditation, and relaxation techniques for carers.	32	31	All services working together in conjunction not ring‐fencing funding.	54	62
Improving inpatient experience for carers, i.e., carers and service users resource pack, open communication and consistent feedback between acute staff and carers.	32	43	Family therapy—patients and their family members (including their carers) receive therapy together.	48	48	NHS services should elevate supporting carers to the same importance as safeguarding.	27	31	Financial grants for travel expenses for carers.	28	25
Staff/leads at hospitals involving and working with carers to develop a full pathway of mental health support. A carers discharge planning involvement and transitional discharge model. Maybe it can be delivered by carers champions within services like IAPT.	41	23	Patient to talk to someone when a carer is not there.	32	33	Tailored peer/social support intervention for carers. Provided in a format that is convenient to the people involved, so they do not become unwell themselves.	41	30	Practical guidance acute‚ community around risk management.	38	49
More time is needed at the end of appointments for staff to speak to carers.	17	33	Basic training/education for patients about specific illnesses and being educated about the importance of more generic stressors that other people may face.	23	31				Understanding co‐ordination of care—who should be doing it, what works, what models are more or less effective.	59	59
									Improving access to therapies for patients.	43	42

Abbreviation: IAPT, improving access to psychological therapies.

For theme 1 ‘Carer Involvement and Improving Carer Experience’, the top three ‘most acceptable’ solutions were (1) having a dedicated family liaison worker or a specialist team to act as a bridge between the hospital and home, (2) starting at admission and identifying the support network for the patient and (3) staff leaders at hospitals working with carers to co‐develop a full pathway of support. The top three most feasible solutions were (1) starting at admission and identifying the support network for the patient, (2) improving inpatient experience for carers, that is, carers and patients resource pack, open communication and consistent feedback between acute staff and carers and (3) more time at the end of appointments for staff to speak to carers.

For theme 2 ‘Patient Wellness and Education’ the top three most acceptable solutions were (1) adapting and implementing existing approaches such as activity scheduling to help patients implement their care plan, (2) patients receiving basic training in self‐care and important coping strategies and (3) basic training/education for patients about specific illnesses as well as generic stressors. The top three most feasible solutions were (1) basic training/education for patients about specific illnesses and being educated about the importance of more generic stressors that other people may face, (2) adapting and implementing existing approaches such as activity scheduling to help the patients plan what they need to do and (3) patients receiving basic training in self‐care and important coping strategies, as the top three, respectively. There is an overlap between the top three solutions for acceptability and feasibility however the order is different.

For theme 3 ‘Carer Wellness and Education’, the top three most acceptable solutions were (1) tailored peer/social support intervention for carers, (2) basic training/education for carers and patient families about specific illnesses and generic stressors and (3) carers needs assessment and mindfulness‐based meditation, and relaxation techniques for carers. The top three most feasible solutions were (1) basic training/education for carers and patient families about specific illnesses and generic stressors; (2a) carers needs assessment and mindfulness‐based meditation, and relaxation techniques for carers; (2b) NHS services and hospital trusts elevating support for carers to the same importance as safeguarding, was ranked as a joint second and (3) tailored peer/social support intervention for carers.

For theme 4 ‘Policy and System Improvements’, the top three most acceptable solutions were (1) improving carer understanding of the co‐ordination of care, (2) all services working together in conjunction not ring‐fencing funding and (3) checking the home environment at admission/predischarge. The top three most feasible solutions were (1) all services working together in conjunction not ring‐fencing funding, (2) improving carer understanding of the co‐ordination of care and (3) checking the home environment at admission/predischarge. Like theme 2, there is an overlap between the top three solutions for acceptability and feasibility however the order is different.

**Figure 2:**
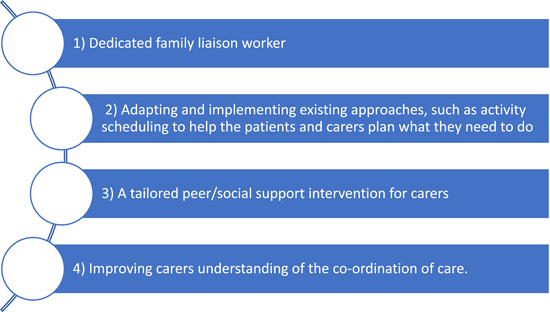
Most feasible and acceptable solutions across all themes.

Across all themes, the top four feasible and acceptable solutions identified by the group were (1) having a dedicated family liaison worker, (2) adapting and implementing existing approaches, such as activity scheduling to help the patients and carers plan what they need to do, (3) a tailored peer/social support intervention for carers and (4) enabling carers to understand the co‐ordination of care (Figure 2).

## 4 DISCUSSION

This study involving a diverse stakeholder group identified many patient safety concerns and carer well‐being risks during care transitions from inpatient mental health hospitals to the community. These include lack of information and support, distress and isolation. Multiple solutions were generated based on the collective knowledge of the diverse stakeholder group; the most highly ranked feasible and acceptable solutions were naming a dedicated family liaison worker and adapting better implementing existing approaches. Four broad themes of solutions were uncovered based on their nature and purpose: carer involvement in improving patient experience, patient wellness and education, carer wellness and education, policy and system improvements.

Our findings are consistent with previous research which highlighted that carers are concerned for their own mental wellbeing and patient safety during this precarious time.^13^ The outcomes of this prioritisation exercise echo previous findings about key problems in relation to patient safety carer wellbeing at transition care points and advance the existing knowledge by identifying stakeholder‐led solutions to these problems. The group felt that providing support to carers of people discharged from mental health hospitals, through intervention development or systems/process/policy change, has the potential to improve patient safety at this particularly distressing time. In line with our findings, carers have previously suggested that there are numerous ways that services could support them.^7^ First, by improving access to information and knowledge about services, systems and medication as well as care plans and self‐care practices. Second, by providing practical support and advocacy support to carers in social/community care services (e.g., through case managers). Third, through promoting self‐awareness, wellbeing and awareness amongst others about the role of carers and patient safety behaviours.^7^

The evidence base on which interventions can effectively improve patient safety and carer well‐being is relatively limited. This is despite carer‐led suggestions and the gradually growing awareness of the need to consistently include carers in care quality and safety improvement programmes for people with mental illness and in parallel support their mental well‐being. A recent systematic review of interventions involving carers in transitions between inpatient mental health hospitals and outpatient care found that three intervention components with increasing levels of complexity have been tested.^21^ They involved psychoeducational sessions in the hospital, structured involvement of carers in discharge planning and follow‐up sessions with patients and carers in community services after discharge, or combinations of these three components in the most complex scenarios of these interventions. Interventions, which included carer participation in discharge planning, appeared to be beneficial in relapse reduction, which is a highly relevant outcome, both clinically and in terms of health care costs.

Evidence suggests that interventions involving carers improved the experience of caring and quality of life amongst people with severe mental illness and reduced the psychological distress of carers^31^ and that intervention components for carers should be considered as part of integrated services for people with severe mental health problems.

Hence, our stakeholder‐led solutions combined with the findings of previous systematic reviews suggest that there is a pressing need for mental health hospitals and community services to adopt strategies to facilitate the implementation of carer involvement for ensuring safe transitional care. We encourage the co‐design of novel carer‐inclusive transitional care interventions informed by the solutions proposed in the current study. In the face of growing evidence showing that the voices of the most vulnerable patients with mental ill health and their carers are often not considered while designing service improvements, we strongly recommend consulting published guidance such as the NIHR quality, diversity, and inclusion strategy while coproducing and testing these solutions as interventions/service improvements. A factorial trial design to test these interventions is recommended to better understand the benefits of individual intervention components on different patient and carer outcomes. The use of this design will also allow for flexible implementation and ensure feasibility within busy services.

### 4.1 Strengths and limitations

One strength of the study was the promotion of open dialogue between diverse stakeholders including carers, academics with different perspectives and health professionals. We also provided the option of a carers‐only group to reduce any potential negative power dynamics and promote inclusivity. As a result, we generated a series of potential intervention ideas that were agreed upon by this diverse group of stakeholders.

However, this study had also some key limitations. The most important limitation is that the attrition rate between the online workshop (phases 1–3) and the final online questionnaire to rank solutions (phase 4) was considerable. We originally planned to complete phase 4 during the workshop, but as there were so many solutions generated the task became too complicated to manage in one session. We, therefore, arranged a follow‐up exercise, which resulted in a moderate attrition rate and incomplete collection of the demographic information of the participants in the workshop. Furthermore, the online nature of the workshop could have resulted in digital exclusion for some carers (especially those who are lacking e‐literacy).

The sampling decision to use established contacts within the study team and social media enabled us to access academic expertise across the three academic fields of knowledge we hoped to combine expertise (Primary Care, Social Care and Public Health). However, in the future, including a greater number of patients and carers and using techniques to access more diverse groups would be beneficial.

### 4.2 Implications and conclusions

The members of our stakeholder group concurred that the transition from mental health hospitals to the community is a particularly distressing period of the care pathway, where patients and carers are particularly vulnerable to safety and well‐being risks. This study identified several feasible and acceptable solutions to enable carers of people transitioning from mental health hospitals into the community to improve patient safety and maintain their own mental wellbeing. Clear policies and financial investments are required to convert these feasible and acceptable solutions into intervention components using a comprehensive co‐production approach. Once co‐produced, these intervention components could be evaluated preferably as a care bundle using a factorial trial design to better understand which components work best for which patient and carer outcomes. This coproduction and evaluation approach would generate crucial knowledge to ensure the longevity and cost‐effectiveness of care‐inclusive transitional care interventions.

## AUTHOR CONTRIBUTIONS

Natasha Tyler and Maria Panagioti devised the design of the study and lead the funding application, lead the workshop and oversaw the project. Sarah McMullen drafted the manuscript, conducted analysis and data collection and planned the logistics of the workshops. Sally Giles and Claire Planner facilitated breakout rooms in the workshop. Ioannis Angelakis applied for ethical approval for the project and devised study documents. Richard N. Keers, Catherine Robinson, Yu Fu and Judith Johnson contributed towards the design of the study and attended the workshop. All authors revised and contributed to the writing of the manuscript.

## CONFLICT OF INTEREST STATEMENT

The authors declare no conflict of interest.

## ETHICS STATEMENT

This study was approved by the University of Manchester Proportionate Ethical Review Panel, reference 2022‐14187‐23633. Informed consent was gained from all participants.

## Data Availability

Data available within the article or its Supporting Information: Materials.
